# Titanium Salan Complexes Displays Strong Antitumor Properties *In Vitro* and *In Vivo* in Mice

**DOI:** 10.1371/journal.pone.0017869

**Published:** 2011-03-21

**Authors:** Timo A. Immel, Ulrich Groth, Thomas Huhn, Peter Öhlschläger

**Affiliations:** 1 Department of Chemistry and Konstanz Research School Chemical Biology, University of Konstanz, Konstanz, Germany; 2 Department of Immunology, University of Konstanz, Konstanz, Germany; Université Joseph Fourier, France

## Abstract

The anticancer activity of titanium complexes has been known since the groundbreaking studies of Köpf and Köpf-Maier on titanocen dichloride. Unfortunately, possibly due to their fast hydrolysis, derivatives of titanocen dichloride failed in clinical studies. Recently, the new family of titanium salan complexes containing tetradentate ONNO ligands with anti-cancer properties has been discovered. These salan complexes are much more stabile in aqueous media. In this study we describe the biological activity of two titanium salan complexes in a mouse model of cervical cancer. High efficiency of this promising complex family was demonstrated for the first time *in vivo*. From these data we conclude that titanium salan complexes display very strong antitumor properties exhibiting only minor side effects. Our results may influence the chemotherapy with metallo therapeutics in the future.

## Introduction

The discovery of the anticancer effects of cisplatin and its clinical introduction in the 1970s represents an important step in the history of the development of cancer drugs. Today, this metal based complex and its next generation analogues, carboplatin and oxaliplatin, play an important role in tumor therapy, but the existing complexes are efficient in only a limited range of cancers and can induce acquired resistance upon treatment [1]. They also often cause severe side-effects like nausea, bone marrow suppression and kidney toxicity [1], [2]. Current research on metal complexes is therefore directed to the investigation of other platinum-based compounds as well as non-platinum metal-based systems with little cross resistance to cisplatin and less side effects [3]–[7]. Among these, titanium(IV)complexes showed encouraging antitumor activity in various cell lines [8]–[10].

Till today, three classes of cytotoxic titanium complexes are known. Most of the complexes investigated so far are derivatives of either titanocene dichloride (Cp_2_TiCl_2_) [11] or diketonato-complexes like budotitane [Ti(bzac)_2_(OEt)_2_; Hbzac  =  Phenylbutane-1,3-dione] [12]. Representatives of these two classes of complexes were tested in clinical trials, but unfortunately, no objective clinical responses were observed [13], [14]. The main disadvantages of these titanium complexes are their fast hydrolysis under physiological conditions: in an aqueous environment, the loss of the labile groups occurs within seconds [15], [16].

With hydrolytic half lives in the range of hours, salan complexes of titanium are much more stable [17]. Exploring this new, third class of titanium complexes with antitumor activity, we could demonstrate that their biological properties can be fine-tuned by varying the substitution pattern of the complexes. The most promising subgroup contains salan complexes bearing halogen residues at the aromatic rings. The IC_50_ values of these halo-salan complexes are comparable to cisplatin and in contrast to their alkyl-substituted counterparts, they almost exclusively induce apoptotic cell death [18]. Moreover, we could demonstrate that these complexes are effective in a cisplatin-resistant cell line [17].

Up to now, few is known about the mechanism of action and cellular uptake of the titanium salan complexes on the one and selectivity and *in vivo* applicability on the other hand. Therefore, we have selected cervical cancer for first preclinical studies. Worldwide approximately 370,000 cases of cervical cancer are being diagnosed each year and almost 200,000 deaths are attributed to this disease [19]. In developed areas the treatment bases mainly on surgery and chemotherapy but nevertheless even when optimal treatment is available about 40% of all cervical cancer patients die of this disease [20]. Therefore, the development of effective and save new anticancer drugs is necessary.

In the current study we analyzed the potency of two titanium salan complexes to induce cell death in C3 cells *in vitro* and tumor regression in C57BL/6 mice.

## Results

As we reported previously, the halogen substituted complexes are highly active against various human cancer cells and nearly exclusively induce apoptotic cell death. Moreover, they display an astonishing hydrolytic stability. Especially complex **2** with a half life of more than 4 days in aqueous media is therefore a promising substance (for an overview of their physicochemical and biological properties see table 1) [17], [18]. The aim of this study was to characterize the activity of two promising titanium salan complexes in more detail and to evaluate their potential clinical value. For this purpose we investigated the biological activity using a broad variety of cancer cell lines *in vitro* and, importantly, the well established C3 mouse model *in vivo*.

**Table 1:** Physicochemical and biological properties of complexes 1 and 2.

properties of complexes 1, 2	complex 1	complex 2
IC_50_ value in Hela S3	1.6±0.1 µM	5.3±0.2 µM
IC_50_ value in Hep G2	2.2±0.2 µM	4.0±0.2 µM
hydrolytic half life	6 h	108 h
apoptotic/dead cells in Hela S3	90.1±4.4%	95.5±2.0036%

IC_50_ values were determined using an AlamarBlue Assay after 48 h of incubation with different concentrations of the complexes. The hydrolytic half life was determined using time resolved ^1^H-NMR. These hydrolysis experiments were conducted in a mixture of 95% [D_8_]THF and 4.8% D_2_O at 37°C. As an internal standard, 0.2% DMSO was added. Cell death was followed via double staining with propidium iodide and fluorescein isothiocyanate-labeled annexin V.

### Titanium salan complexes were synthesized in high yields

**Figure 1:**
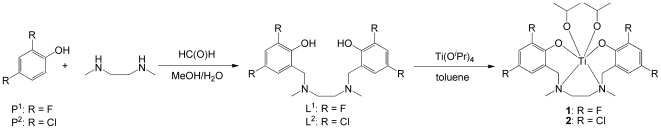
Synthesis of the salan ligands and titanium salan complexes. The halo-salan ligands L^1^ and L^2^ were synthesised *via* a Mannich-condensation of the respective phenol, formaldehyde and *N,N*'-ethylenediamine. The ligands were then metallated with Ti(O*^i^*Pr)4 in toluene to give complexes **1** and **2**.

Complexes **1** and **2** were accessible by refluxing the appropriate phenol N,N'-dialkylethylenediamine, and formaldehyde in methanol and metallating the resulting ligands with titanium tetraisopropoxide (Ti-(O*^i^*Pr)_4_) [18]. To remove trace impurities, the complexes were recrystallized at least once from *n*-hexane or toluene and purities were proven by combustion analysis (for details see materials and methods). This elegant two step synthesis allowed for the preparation of the complexes in high yields (more than 10 g, respectively) and purity (Figure 1).

### Complexes 1 and 2 are able to suppress cell proliferation in the murine C3 model of cervical cancer but also in various human cancer cells *in vitro*

**Figure 2:**
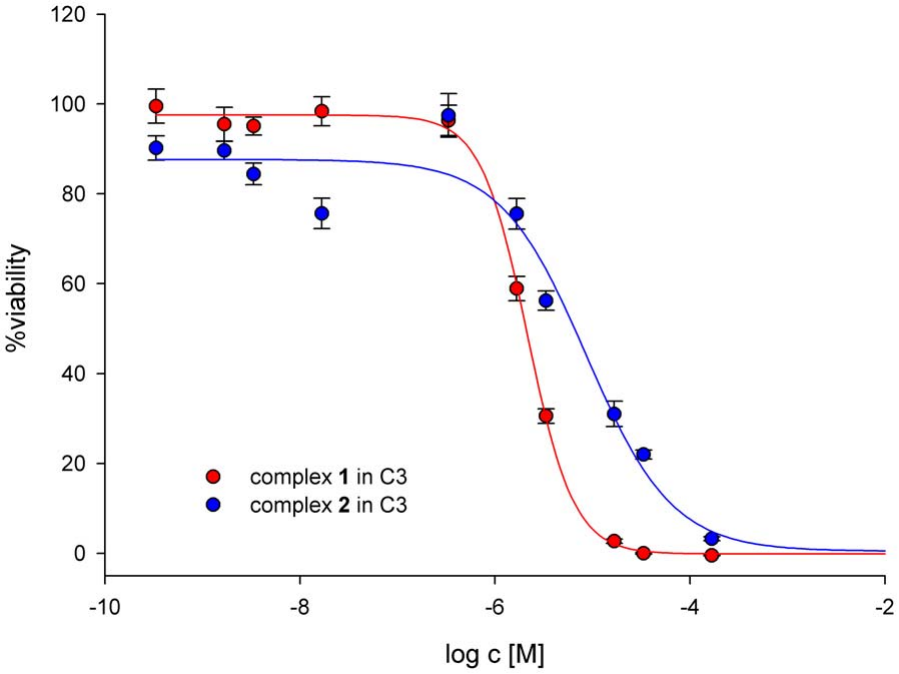
Viability of C3 cells after incubation for 48 h with complexes 1 and 2. Dose-response curves were obtained using an AlamarBlue assay. Measuring the fluorescence and comparison to a negative control gave the relative amount of cells surviving the treatment. The shown dose-effect curves result from three independent test series done in four replicates.

To test efficacy of complexes **1** and **2** in C3 cells, both compounds were tested using the well-established AlamarBlue assay. This assay commonly used to determine cell proliferation is known to be highly reproducible and more sensitive than the MTT assay. The obtained dose-activity curves for complexes **1** and **2** in C3 cells are shown in Figure 2. With an IC_50_ value of 2.2±0.1 µM complex **1** was highly active in C3 cells. In fact, it represents one of the most active titanium salan complexes found so far. Complex **2** was slightly less toxic, the IC_50_ value was 8.6±1.3 µM (Figure 2). Both IC_50_ values are given as mean values from at least three independent experiments each done in four replicates. These IC_50_-values are comparable to those found for these complexes in the human cancer cell lines HeLa S3 and Hep G2.

To further show the broad applicability of these halogen substituted titan salan complexes, complex **1**, representing the most active complex, was sent to the US National Cancer Institute (NCI) for testing its activity in 57 human tumor cell lines (NCI60 anticancer drug screen). In 17 cell lines the complex was only slightly active with an IC_50_ value of more than 100 µM. In particular, it was not toxic to every of the leukemia cell lines tested so far. In further 20 cell lines, the complex showed a mediocre activity between 10 and 100 µM. In 19 tumor cell lines, the complex was highly active with IC_50_ values below 10 µM. Especial melanoma cells seem to be extremely sensitive to titanium salan complexes. Together, we conclude that complex **1** suppresses tumor cell proliferation *in vitro* in more than two thirds of all cell lines investigated (Table 2, 3, and 4). The complete data are shown in the supporting information (Figure S1).

**Table 2:** IC_50_-values of titanium salan complex 1 in Breast-, Colon-, and CNS human cancer cell lines.

Breast Cancer	IC_50_	Colon Cancer	IC_50_	CNS Cancer	IC_50_
MCF7	>100 µM	COLO	46.3 µM	SF-268	7.4 µM
MDA-MB-231/ATCC	>100 µM	HCT-116	29.2 µM	SF-295	6.0 µM
HS 578T	>100 µM	HCT-15	>100 µM	SF-539	>100 µM
BT-549	32.5 µM	HT29	8.7 µM	SNB-19	>100 µM
T-47D	60.1 µM	KM12	17.6 µM	SNB-75	8.8 µM
MDA-MB-468	9.9 µM	SW-620	9.4 µM	U251	4.6 µM

Cells were cultivated in 96 well plates and incubated with a dilution series containing five different concentrations of complex **1**. The cells were fixed, stained with sulforhodamine B and compared to a control. From the dose-response curves (see supplements, Figure S1), the IC_50_-values were calculated. The full test protocol is available under http://dtp.nci.nih.gov/branches/btb/ivclsp.html.

**Table 3:** IC_50_-values of titanium salan complex 1 in Melanoma-, Lung-, and Renal human cancer cell lines.

Melanoma	IC_50_	Lung Cancer	IC_50_	Renal Cancer	IC_50_
LOX IMVI	6.5 µM	A549/ATCC	39.8 µM	786-0	14.8 µM
MALME-3M	4.3 µM	EKVX	59.5 µM	A498	6.8 µM
M14	6.7 µM	HOP-92	3.6 µM	ACHN	>100 µM
MDA-MB-435	5.7 µM	NCI-H226	>100 µM	CAKI-1	44.9 µM
SK-MEL-2	37.6 µM	NCI-H23	6.6 µM	RXF	4.4 µM
SK-MEL-28	5.4 µM	NCI-H322M	20.3 µM	SN12C	43.3 µM
SK-MEL-5	4.3 µM	NCI-H460	30.9 µM	TK-10	>100 µM
UACC-257	12.6 µM	NCI-H522	5.3 µM	UO-31	24.2 µM
UACC-62	>100 µM				

Conditions of treatment, see table 2.

**Table 4:** IC_50_-values of titanium salan complex 1 in Ovarian-, Leukemia-, and Prostate human cancer cell lines.

Ovarian Cancer	IC_50_	Leukemia	IC_50_	Prostate Cancer	IC_50_
IGROV1	6.2 µM	CCRF-CEM	>100 µM	PC-3	28.6 µM
OVCAR-3	4.6 µM	HL-60(TB)	>100 µM	DU-145	21.0 µM
OVCAR-4	78.7 µM	K-562	>100 µM		
OVCAR-5	57.2 µM	MOLT-4	>100 µM		
OVCAR-8	>100 µM	SR	>100 µM		
NCI/ADR-RES	>100 µM				
SK-OV-3	59.1 µM				

Conditions of treatment, see table 2.

.

### Both titanium salan complexes are well tolerated in animals up to single doses of 25 mg/kg

In a first experiment we investigated the tolerance of titanium salan complexes **1** and **2** in direct comparison to conventional cisplatin in animals. Thereby, we hypothesized that the complexes are much more tolerated in direct comparison to cisplatin and decided to compare a well established dose of cisplatin in the murine model with different doses of the compounds. Mice (n = 6/group) received 2 mg/kg cisplatin [21] or once 50, 25, 12.5 or 5 mg/kg of the complexes **1** and **2**, respectively. Noteworthy, after 30 days all mice of the cisplatin group and all animals of the 50 and 25 mg/kg groups (complex **1** and **2**, respectively) displayed clear signs of side effects like hair loss, apathy up to death (data not shown). Interestingly, all animals of the other complex-treated groups (25, 12, 5 mg/kg, respectively) survived and no fatal side effects were observed by eye. From this outcome we decided to test first a 30 mg/kg dose given at multiple applications (m.a.) (5 mg/kg on days 0, 2, 4, 7, 9, 11, respectively [30 mg/kg m.a. group]) *versus* a 50 mg/kg dose also given at m.a. (30 mg/kg on day 0 and 20 mg/kg on day 9 [50 mg/kg m.a. group]) treatment regime in the next set of experiments.

### Titanium salan complexes mediate tumor regression in a transplantable mouse tumor model for cervical cancer

We were interested to investigate the therapeutic *in vitro* effects of the two titanium salan complexes and took advantage of the well established C3 tumor model of cervical cancer [22]. After animals (n = 10/group) had developed palpable tumors (2–4 mm^2^ in average) treatment was started (day 0).

Two groups received 30 mg/kg at m.a. (days 0, 2, 4, 7, 9, 11) and control animals received DMSO/PBS/Tween in the same time scale. We observed a significantly reduced tumor growth in the 30 mg/kg m.a. group that received complex **1** (122±23 mm^2^ *vs* 240±32 mm^2^ in the control group, p-value: 0.008) at day 31 whereas the effect was dramatically enhanced within the 30 mg/kg m.a. dose group treated with complex **2** (34±12 mm^2^, p-value to the control group: 0.0001, p-value to the 30 mg/kg m.a. complex **1** group: 0.03) (table 5).

**Table 5:** Growth of established C3 tumors in C57BL/6 mice after treatment with titanium salan complexes 1, 2.

	average tumor size at day 0 (mm^2^) ± S.E.M	average tumor size at day 31 (mm^2^) ± S.E.M	number of total regressors at day 31
complex **1** * (30 mg/kg m.a.)	3±2	122±23	0
complex **2** a* (30 mg/kg m.a.)	3±1	34±12	0
complex **1** ^#^(50 mg/kg m.a.)	2±1	12±8	2
complex **2** ^#^ (50 mg/kg m.a.)	4±2	11±6	3
DMSO/PBS/0.5% Tween 80	2±1	240±32	0

Mice received tumor cells s.c. into the flank and were treated either with 30 mg/kg at multiple applications (m.a.) (days 0, 2, 4, 7, 9, 11 with 5 mg/kg, respectively) (*) or with 50 mg/kg at m.a. (day 0: 30 mg/kg and day 9: 20 mg/kg) (^#^). Data gives the mean ± SEM at days 0 and 31 and the number of completely tumor free animals. Because of the size of tumors in the control group the experiment was terminated at day 31. One representative of two experiments is shown.

In order to determine the effects of a 50 mg/kg dose regime combined with a lower application frequency on the tumor growth, two further groups were treated with 30 mg/kg (day 0) followed by an injection of 20 mg/kg (day 9) with the respective titanium salan complex (50 mg/kg m.a.). Compared to the control group (DMSO/PBS/Tween) both compounds mediate significant tumor regression (p-values for both complexes: 0.0001).

Interestingly, no total tumor regressors were found within the 30 mg/kg m.a. groups. In contrast, 2 total tumor regressors (no tumor palpable) were documented in the 50 mg/kg complex **1** group (tumor size: 12±8 mm^2^) and 3 total regressors were observed within the complex **2** treated animals (tumor size: 11±6 mm^2^). In direct comparison of the 30 mg/kg *vs* the 50 mg/kg groups, both complexes are more effective at higher doses (p-values: 0.0003 for complex **1** and 0.1 for complex 2) (table 5).

But in contrast to the 30 mg/kg m.a. application regime side effects like alopecia and apathy were observed in all animals treated with 50 mg/kg, especially in the complex **1** group (data not shown). From this set of experiments we conclude that titanium salan complex **2** is well tolerated and highly active *in vitro* at 30 mg/kg dose given at m.a.

Next, we wanted to know if the observed therapeutic tumor effect of the well tolerated complex **2** (30 mg/kg at m.a.) could be enhanced without strengthen any side effects. Therefore, tumor-bearing animals (n = 10/group) were treated either with 50 mg/kg at m.a. (day 0: 30 mg/kg, day 7: 20 mg/kg) or with a single dose of 30 mg/kg (day 0). Again, control animals received DMSO/PBS/Tween (days 0, 7) and reached at day 29 a tumor size of 268±24 mm^2^. A comparable therapeutic effect as in the first set of *in vivo* experiments was observed in the 50 mg/kg m.a. group (tumor size: 12±3 mm^2^, p-value to the control: <0.0001) (see table 6). As mentioned above, side effects like alopecia and apathy were observed. In contrast, animals of the 30 mg/kg s.a. group developed significant larger tumors (64±9 mm^2^, p-value to the 50 mg/kg m.a. group: <0.0001) and no alopecia but mild apathy were observed. A comparison of the 30 mg/kg m.a. (table 5) with the 30 mg/kg s.a. (table 6) reveals higher efficacy if the drug is applied distributed over 11 days (p-value: 0.06). The single application (s.a.) of 30 mg/kg of complex **2** is therefore less effective than the application of the same complex amount at multiple days.

**Table 6:** Growth of established C3 tumors in C57BL/6 mice after treatment with titanium salan complexes 1, 2.

	average tumor size at day 0 (mm^2^) ± S.E.M	average tumor size at day 29 (mm^2^) ± S.E.M	number of total regressors at day 29
complex **2** ^#^ (30 mg/kg s.a.)	2±1	64±9	0
complex **2** ^*^ (50 mg/kg m.a.)	2±1	12±3	2
DMSO/PBS/0.5% Tween 80	3±2	268±24	0

Mice received tumor cells s.c. into the flank and were treated either with 50 mg/kg at multiple applications (m.a.) (day 0: 30 mg/kg and day 7: 20 mg/kg) (*) or with a single application (s.a.) of 30 mg/kg on day 0 (**^#^**). Data gives the mean ± SEM at days 0 and 29 and the number of completely tumor free animals. Because of the size of tumors in the control group the experiment was terminated at day 29. One representative of two experiments is shown.

Together, these experiments imply that the titanium salan complex **2** combines strong antitumor effects *in vitro* and *in vivo* and is highly tolerated when given at low doses distributed over multiple injections.

## Discussion

In this study we were able to confirm the cytotoxicity of two titanium salan complexes in various cell lines and showed for the first time the activity of this class of complexes *in vivo*. We were able to demonstrate that one out of two complexes investigated in this study combines high antitumoral effects with a low adverse reaction profile.

Titanium salan complexes represent a promising group of chemotherapeutic agents, belonging to the class of metallo drugs. Cisplatin as the first metallo therapeutic agent used in chemotherapy was first synthesized in the nineteenth century but clinical application did not commence until the 1970s. After this major landmark in the history of successful drugs for the treatment of cancer, carboplatin as a safer second generation analogue was introduced approximately ten years later. Next, the development of improved analogues slowed for many years but recently the interest in well tolerated and effective metallo therapeutic compounds has increased. Aiming for more effective and less toxic substances, titanium complexes attracted attention. Herein, we could show that such titanium complexes are effective in a variety of cancer cells. In tests on 57 human cancer cell lines, titanium salan complexes showed mediocre activity in 20 cell lines and high activity with IC_50_ values below 10 µM in 19 tumor cell lines. These results encouraged us to further investigate the applicability of these complexes as anti cancer drugs. As we decided to use the well established mouse C3 model for cervical cancer, we tested the activity of our complexes against C3 cells *in vitro* and, more importantly, *in vivo*.

Indeed, cervical cancer is one potential field of application for the titanium salan complexes due to the fact that survival in women with recurrent or metastatic cervical cancer remains poor and therefore, more effective and less toxic regimes are needed. The superiority of the complex **2** in contrast to the complex **1** counterpart is especially interesting because **1** showed a nearly four fold higher activity against C3 cells *in vitro*. This observation could be explained by the much higher stability of complex **2** in aqueous media. With a half life of 106 h, this complex is nearly 20 times as stabile as its fluoro counterpart complex **1**. It seems that a hydrolytic detoxification of complex **1** leads to a lower effective dose of the active substance on the one hand. On the other hand, evolving toxic products from the hydrolysis process might lead to the more pronounced side effects observed with complex **1**. In fact, the titanium salan complex **2** mediates strong suppression of the tumor growth even when administered at relative low doses and combines this feature at this dosage with outstanding tolerance. Interestingly, the repeated low dose application regime (six times 5 mg/kg, respectively) was most effective which could be explained by prolonged *in vivo* drug availability.

Together, the compound **2** holds promise for a more effective anticancer treatment approach in humans. This is especially true as we could show, that this complex is highly active against cisplatin resistant human bladder cancer cell line MGH-U1 and a cross resistance to cisplatin is not observed [17]. These results will also influence the search of new titanium complexes. A further improvement of the stability in aqueous media may for example be achieved by varying the labile ligands. In the case of titanocene derivatives, this strategy has already been successful [23].

Because we are experienced in the development of therapeutic vaccines against cervical cancer [24]–[27] we will next combine immunotherapy time-shifted to treatment intervals with the titanium salan complex **2**. A multidisciplinary treatment approach could significantly improve the outcome of further cancer therapies and indeed, synergistic effects of combined anticancer therapies are described [28].

## Materials and Methods

### Synthesis of the ligands

Synthesis of the complexes was conducted using standard Schlenk technique, all solvents were purified according to standard procedures. NMR spectra were recorded on a JEOL Eclipse 400 or a Bruker Avance DRX 600 spectrometer. Structure assignments were done based on 2D NMR experiments (COSY, HMBC, HSQC). Elemental analyses were performed at the microanalytical laboratory of the University of Constance.

The respective (20 mmol) 2,4-disubstituted phenol was suspended in methanol (10 ml) and formaldehyde solution (7.5 ml, 36%) and N,N'-dimethylethylene diamine (10 mmol) were added. The mixture was refluxed for 24 h and allowed to cool to room temperature. Upon cooling to 6°C, the products crystallized as colorless solids.

L^1^: ^1^H-NMR (600 MHz, CDCl_3_): δ = 6.76 (ddd, J = 11.2 Hz, 8.5 Hz, 2.6 Hz, 2H, H_ar_), 6.50 (d, J = 8.5 Hz, 2H, H_ar_), 3.70 (s, 4H, NCH_2_C_ar_), 2.69 (s, 4H, NCH_2_CH_2_N), 2.32 ppm (s, 6H, NCH_3_). ^13^C-NMR (151 MHz, CDCl_3_): δ = 154.7 (dd, J = 239.5 Hz, 11.0 Hz, FC_ar_), 150.7 (dd, J = 246.8 Hz, 12.1 Hz, FC_ar_), 142.0 (dd, J = 12.4 Hz, 3.3 Hz, CH_2_ C_ar_), 123.8 (dd, J = 8.3 Hz, 4.0 Hz, OC_ar_), 109.9 (dd, J = 23.0 Hz, 3.5 Hz, HC_ar_), 103.9 (dd, 26.4 Hz, 22.2 Hz, HC_ar_), 61.2 (NCH_2_C_ar_), 54.1 (NCH_2_CH_2_N), 41.8 ppm (NCH_3_). Elemental analysis calcd (%) for C_18_H_20_F_4_N_2_O_2_: C: 58.06; H: 5.41; N: 7.52, found: C: 57.98; H: 5.40; N: 7.60. L^2^: ^1^H-NMR (400 MHz, CDCl_3_): δ = 7.27 (d, J = 2.5 Hz, 2H, H_ar_), 6.87 (d, J = 2.5 Hz, 2H, H_ar_), 3.70 (s, 4H, NCH_2_C_ar_), 2.72 (s, 4H, NCH_2_CH_2_N), 2.33 ppm (s, 6H, NCH_3_). ^13^C-NMR (101 MHz, CDCl_3_): δ = 152.4 (C_ar_), 129.0 (C_ar_), 126.8 (C_ar_), 123.7 (C_ar_), 123.4 (C_ar_), 121.7 (C_ar_), 61.0 (NCH_2_C_ar_), 54.0 (NCH_2_CH_2_N), 41.8 ppm (NCH_3_). Elemental analysis calcd (%) for C_18_H_20_Cl_4_N_2_O_2_: C: 49.34; H: 4.60; N: 6.39, found: C: 49.38; H: 4.51; N: 6.33.

### Synthesis of the complexes

The ligands (3.9 mmol) were dissolved in toluene (20 ml) and Ti(O*^i^*Pr)_4_ (3.9 mmol, 420 mg, 860 µl) was added under nitrogen atmosphere. The yellow reaction mixture was allowed to stir over night at room temperature. The solvent was removed under reduced pressure and the obtained yellow solid was recrystallized to give complexes **1**, **2**.

**1**:^ 1^H-NMR (600 MHz, CDCl_3_): δ = 6.78–6.72 (m, 2H, H_ar_), 6.52–6.48 (m, 2H, H_ar_), 5.09 (sept., J = 6.1 Hz, 2H, TiOCH), 4.62 (d, J = 13.7 Hz, 2H, NCH_2_C_ar_), 3.15 (d, J = 13.7 Hz, 2H, NCH_2_C_ar_), 2.97 (d, J = 9.4 Hz, 2H, NCH_2_CH_2_N), 2.46 (s, 6H, 2x NCH_3_), 1.90 (d, J = 9.4 Hz, 2H, NCH_2_CH_2_N), 1.28 (d, J = 6.2 Hz, 6H, CH(CH_3_)_2_), 1.24 ppm (d, J = 6.2 Hz, 6H, CH(CH_3_)_2_). ^13^C-NMR (151 MHz, CDCl_3_): δ = 153.3 (dd, J = 237.1 Hz, 11.2 Hz, FC_ar_), 150.6 (dd, J = 246.3 Hz, 12.4 Hz, FC_ar_), 146.5 (dd, J = 12.4 Hz, 2.8 Hz, CC_ar_), 126.0 (dd, J = 8.5 Hz, 4.1 Hz, OC_ar_), 110.4 (dd, J = 22.5 Hz, 3.3 Hz, HC_ar_), 103.7 (dd, 26.1 Hz, 22.5 Hz, HC_ar4_), 78.9 (TiOCH), 63.7 (dd, J = 2.8 Hz, 2.2 Hz, NCH_2_C_ar_), 51.8 (NCH_2_CH_2_N), 46.9 (NCH_3_), 25.8 (CH(CH_3_)_2_), 25.4 ppm CH(CH_3_)_2_). Elemental analysis calcd (%) for C_24_H_32_F_4_N_2_O_4_Ti: C 53.74, H 6.01, N 5.22; found: C 53.79, H 5.89, N 5.14. **2**: ^1^H-NMR (600 MHz, CDCl_3_): δ = 7.28 (d, *J* = 2.6 Hz, 2H, H_ar_), 6.86 (d, *J* = 2.6 Hz, 2H, H_ar_), 5.21 (sept., *J* = 6.1 Hz, 2H, TiOCH), 4.63 (d, *J* = 13.6 Hz, 2H, NCH_2_C_ar_), 3.14 (d, *J* = 13.6 Hz, 2H, NCH_2_C_ar_), 2.91 (d, *J* = 9.4 Hz, 2H, NCH_2_CH_2_N), 2.44 (s, 6H, 2x NCH_3_), 1.88 (d, *J* = 9.4 Hz, 2H, NCH_2_CH_2_N), 1.30 (d, *J* = 6.2 Hz, 6H, CH(CH_3_)_2_), 1.23 ppm (d, *J* = 6.2 Hz, 6H, CH(CH_3_)_2_). ^13^C-NMR (151 MHz, CDCl_3_): δ = 156.2 (C_ar_), 129.0 (C_ar_), 127.6 (C_ar_), 126.1 (C_ar_), 122.6 (C_ar_), 121.1 (C_ar_), 79.2 (TiOCH), 63.8 (NCH_2_C_ar_), 51.7 (NCH_2_CH_2_N), 47.1 (NCH_3_), 26.0 (CH(CH_3_)_2_), 25.6 ppm (CH(CH_3_)_2_). Elemental analysis calcd (%) for C_24_H_32_Cl_4_N_2_O_4_Ti: C 47.87, H 5.36, N 4.65; found: C 47.97, H 5.46, N 4.68.

### Cell culture

C3 tumor cells (C57BL/6 origin) derived from embryonic mouse cells transfected with the complete HPV-16 genome [22] were cultured in RPMI 1640 supplemented with heat-inactivated 5% (v/v) fetal calf serum (FCS, Gibco, Eggenstein, Germany), 2 mM L-glutamine, penicillin (100 U/ml), streptomycin (100 µg/ml) and kanamycin (0.1 mg/ml) at 37°C in humidified 5% CO_2_ atmosphere. Cells were split every three days and tested for mycoplasma infections using a mycoplasma detection kit (Roche Applied Science).

### AlamarBlue assay with C3 cells

AlamarBlue (BioSource Europe), the dark blue colored sodium salt of resazurin (7-Hydroxy-3H-phenoxazin-3-one-10-oxide) was used to measure growth and viability of cells which are capable of reducing it to the fluorescent, pink colored resorufin (7-Hydroxy-3H-phenoxazin-3-one) [29]. Cells were seeded in 96-well plates (1.8×10^4^ cells/well) and allowed to attach for 24 h. Complexes to be tested were dissolved in a suitable amount of DMSO. Different concentrations were prepared by serial dilution with medium to give final concentrations with a maximum DMSO content of 1%. The cells were then incubated for 48 h with 100 µl each of above dilution series. AlamarBlue (10 µl) was added and the cells were incubated for another hour. After excitation at 530 nm, fluorescence at 590 nm was measured using a FL600 Fluorescence Microplate Reader (Bio-TEK). Cell viability is expressed in percent with respect to a control containing only pure medium and 1% DMSO incubated under identical conditions. All experiments were repeated for a minimum of three times with each experiment done in four replicates. The resulting curves were fitted using Sigma plot 10.0 [30].

### Tumor regression studies

All animal work has been conducted according to relevant national and international guidelines. The animal studies were reviewed and approved by the local ethical committee (Regierungspräsidium Freiburg, Freiburg, Germany, approval number: 35/9185.81/G-06/48 (2008), permission given to P. Öhlschläger). C57BL/6 mice received 0.5×10^6^ HPV-16 E7 expressing C3 [22] cells in 100 µl of PBS subcutaneously in the right shaved flank (needles: 20G 1½” BD Microlance 3). When small tumors were palpable in all animals (days 9–15) the first treatment (complexes **1** and **2**, respectively) was applied intraperitoneally (i.p.) in 100 µl DMSO/PBS/0.5% Tween 80 and control animals received DMSO/PBS/0.5% Tween 80 only. The treatment was repeated as mentioned up to five times. Tumor sizes were measured with a caliper and were determined every 2–4 days until mice had to be sacrificed (tumor size of 400 mm^2^ or when tumors were bleeding). Tumor sizes of the mice within a group were calculated as arithmetic means with standard error of the means (SEMs). All operations on live animals were performed under Isoflurane anesthesia (CuraMed Pharma, Karlsruhe, Germany).

### Statistical analysis

Differences of means between experimental and control group were considered statistically significant when p was <0.05 by unpaired Students t-test.

## Supporting Information

Figure S1 **IC_50_-values of titanium salan complex 1 in different human cancer cell lines.** Dose response curves of complex **1** in 57 different human cancer cell lines. The data show the broad spectrum of activity of titanium salan complexes.
(TIF)
