# Supplementary figures and images for: Titanium Salan Complexes Displays Strong Antitumor Properties In Vitro and In Vivo in Mice
Source: PLoS One. 2011 Mar 21;6(3):e17869. doi: 10.1371/journal.pone.0017869 (PMC3061874; doi:10.1371/journal.pone.0017869)

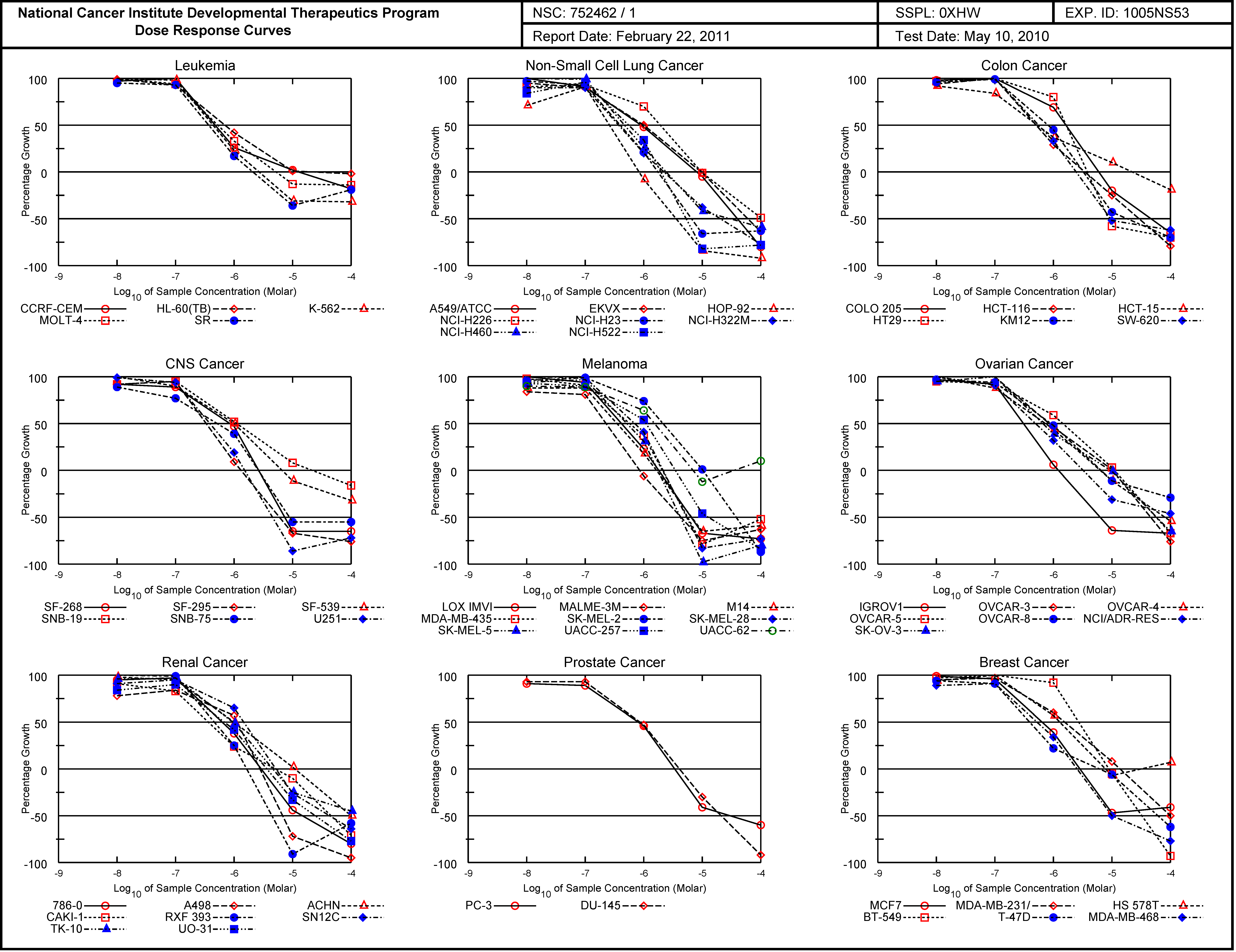

Supplement: Figure S1 — IC50-values of titanium salan complex 1 in different human cancer cell lines. Dose response curves of complex 1 in 57 different human cancer cell lines. The data show the broad spectrum of activity of titanium salan complexes. (TIF) [file pone.0017869.s001.tif]
